# Impact of platelet transfusion refractoriness in the first 30 days post-hematopoietic stem cell transplantation on outcomes of patients with myelodysplastic syndrome

**DOI:** 10.3389/fimmu.2024.1437176

**Published:** 2024-09-25

**Authors:** Yuanfeng Zhang, Yan Wang, Runzhi Ma, Li Liu, Jiali Sun, Xin Chen, Donglin Yang, Aiming Pang, Rongli Zhang, Qiaoling Ma, Weihua Zhai, Yi He, Jialin Wei, Tingting Zhang, Erlie Jiang, MingZhe Han, Sizhou Feng

**Affiliations:** ^1^ State Key Laboratory of Experimental Hematology, National Clinical Research Center for Blood Diseases, Institute of Hematology and Blood Diseases Hospital, Chinese Academy of Medical Sciences and Peking Union Medical College, Tianjin, China; ^2^ Department of Hematology, The Affiliated Yantai Yuhuangding Hospital of Qingdao University, Yantai, Shandong, China

**Keywords:** myelodysplastic syndrome, transplantation, platelet transfusion refractoriness, stem, cell

## Abstract

**Introduction:**

Currently, no study has determined whether platelet transfusion refractoriness (PTR) post-hematopoietic stem cell transplantation (HSCT) before engraftment in patients with myelodysplastic syndrome (MDS) would impacts clinical outcomes.

**Methods:**

We performed a MDS-specific retrospective analysis to determine whether PTR in one-month post-HSCT in patients with MDS could influence outcomes.

**Results and discussion:**

Among the 315 patients enrolled, 110 (34.9 %) had PTR from stem cell infusion to one-month post-HSCT. Baseline characteristics of the PTR and non-PTR groups were similar. We found that patients with PTR had a slower and lower rate of platelet engraftment by day 28, as well as a slower recovery of neutrophils. The median days of neutrophil and platelet engraftment were 14 days (9-23) and 17 days (8-28) in the PTR groups versus 13 days (9-23) and 15 days (7-28) in the non-PTR group (P<0.001). By day 28, 84 of 110 patients (76.4%) with PTR achieved platelet engraftment compared with 181 of 205 patients (88.3%) without PTR achieving platelet engraftment (P=0.007). In addition, patients in the PTR group received significantly more red blood cell (median, 17 units vs. 10 units, P<0.001) and platelet transfusions (median, 13 units vs. 7 units, P<0.001). However, the overall survival was similar between the two groups. PTR in one-month post-HSCT, haploidentical donor, and ferritin level>1041ng/ml (median level) were independent adverse factors of platelet engraftment.

## Introduction

Myelodysplastic syndrome (MDS) is a malignant hematologic disease in adults characterized by cytopenia and a high risk of transformation into acute leukemia in certain subgroups (1). Allogeneic hematopoietic stem cell transplantation (allo-HSCT) is the only curative therapy available for these patients. Platelet transfusion refractoriness (PTR) is defined as an inadequate increase in platelet count after transfusion that may impair the survival of patients undergoing allo-HSCT (2–6). MDS patients usually experience heavy transfusion dependence before transplantation, which may contribute to a high PTR rate (7). However, different mechanisms may lead to PTR before and after allo-HSCT (8). For instance, non-immune-mediated factors, rather than immune-mediated factors, mainly play a role in post-HSCT PTR (5). The reason for this phenomenon may partially be that hematologic recovery after HSCT is from the donor. Currently, no study has determined whether PTR post-HSCT before engraftment in patients would impacts clinical outcomes. Therefore, we performed this retrospective study to compare the outcomes of MDS patients with or without PTR post-HSCT in one month over the same period of investigation.

## Patients and methods

### Patients

Between 2010 and July 2023, 340 patients with MDS or MSD/MPN, who consecutively underwent allo-HSCT, were screened. The enrollment criteria included patients diagnosed with MDS or MDS/MPN according to the fourth edition of WHO who underwent allo-HSCT during this period (1). The exclusion criteria included patients with incomplete data (N=5), those who died (N=6), or those not evaluated for PTR (N=14) at one-month post-HSCT. Finally, 315 patients were enrolled in this study. This study was approved by the Ethics Committee of our hospital (IIT2021011-EC-1).

### Conditioning regimen and transplantation procedure

The conditioning regimen and graft-versus-host (GvHD) prophylaxis were determined by physicians. The backbone protocol was a myeloablative conditioning regimen composed of busulfan 3.2 mg/kg/day for three days, cyclophosphamide 40 mg/kg/day for two days, fludarabine 30 mg/m^2^/day for three days, and cytarabine 2-4g/m^2^/day at divided dose for three days (9). Fludarabine and cytarabine were replaced with cladribine at 5mg/m^2^/day and idarubicin at 12 mg/m^2^/day for three days, respectively. In some cases, we also added 5-day decitabine to the conditioning (10). Patients with a matched unrelated donor (MUD) or haploidentical donor (HID) received rabbit anti-thymocyte globulin (ATG) at 2.5 mg/kg/day of 4 days. Low-dose methotrexate (MTX, 15 mg/m^2^ on day +1 and 10 mg/m^2^ on days +3, +6, and +11) and tacrolimus or cyclosporine were administered for the prevention of acute graft versus host disease (aGvHD). A regimen of tacrolimus or cyclosporine, short-term MTX, and mycophenolate mofetil (MMF) was administered to patients who underwent transplantation from a MUD or HID (**Supplementary Table S1**). Antibacterial, antifungal, and antiviral prophylactic agents were administered according to our protocol (11).

### Definitions

Neutrophil and platelet engraftment (12), aGvHD (13), chronic GvHD (cGvHD) (14), graft failure (GF) (15), and primary cause of death (COD) (16) were defined based on previously reported criteria. In our study, the platelet transfusion protocol is single-donor (apheresis) products, and a 12-hour corrected count increment (CCI) was calculated 12h after platelet transfusion with the following formula: (12h post-transfusion platelet count/μL−pre-transfusion platelet count/μL) ×body surface area/2.5[×10^11^] (mean number of platelets transfused in our center). PTR was defined as a CCI<5×10^9^/L on two sequential occasions (5, 17). Therapy-related mortality (TRM) was defined as death without relapse. Treatment failure after HSCT was defined as death, relapse, or GF, whichever occurred first. Disease-free survival (DFS) was defined as the time from the second month post-HSCT to treatment failure or last follow-up. Overall survival (OS) was defined as the time from the second month post-HSCT to death or last follow-up.

### Statistical analysis

The primary objective of this study was to compare the major outcomes of patients with MDS who underwent allo-HSCT with or without PTR in one-month post-HSCT.

All patients attended an outpatient department or telephone follow-up. The final follow-up date was in July 2023. Continuous and categorical variables were compared using the Mann–Whitney U test, chi-square test, and Fisher’s exact test, respectively. The median follow-up of patients was calculated using the reverse Kaplan–Meier method. The cumulative incidences (CI) of GvHD and TRM were estimated using the competing risk model and compared using Gray’s test. Death was considered as a competing event for GvHD. The probabilities of OS and DFS were estimated using the Kaplan–Meier method, and differences were compared using the log–rank test. Variables with *P* values ≤0.05 in the univariate analysis were entered in multivariate analyses which were used to identify factors impacting engraftment. We used R software packages (R 4.0.5), GraphPad Prism 5, and SPSS 25.0, to perform the statistical analyses. Figures were generated using GraphPad Prism 5. All *P* values were two-sided, and the results were considered statistically significant at *P <*0.05.

## Results

### Characteristic of patients

The basic characteristics of the enrolled patients are summarized in **Table 1**. In short, among the 315 patients analyzed, 110 (34.9%) were diagnosed with PTR in one-month post-HSCT. The median age of the patients in the two groups was comparable (45 vs. 45 years, *P*=0.521). Remarkably, 64 patients and 102 patients had received platelet transfusion twice or more before transplantation in the PTR and non-PTR group, respectively. Among those patients, 41 (64.06%) and 27 (26.47%) patients experienced PTR pre-HSCT with significant difference between the two groups (*P*<0.001). There were no differences in terms of patient sex, diagnosis, pre-HSCT ferritin level, donor age, donor source, donor-recipient relationship, donor-patient sex match, blood types of donors to recipients, or conditioning regimen between the two groups. In addition, the numbers of infused mononuclear and CD34^+^ cells were not significantly different between the two groups. In addition, anti-HLA antibodies result before-HSCT were available in 38, 92, and 6 patients of MSD, HID, and MUD donors, respectively. Among those patients, 12 (31.58%), 26 (28.26%), and 2 (33.33%) patients were positive for anti-HLA I antibodies, respectively. Of the 25 patients tested for HPA and other antibodies before HSCT, 18 were negative, while 4 were positive for IIb/IIIa antibodies, 2 were positive forIa/IIa, and one was positive for both IIb/IIIa andIa/IIa. The median duration of refractoriness was 13 days (range: 3 to 232), and only 7 patients suffered PTR after engraftment.

**Table 1:** Baseline characteristics of patients.

Variables	Factors	Patients with PTR (N=110)	Patients without PTR (N=205)	*P* value
Patient age, years, median (range)	Continuous variable	45 (15-61)	45 (12-63)	0.521
Patient gender, no. (%)	Male	79 (71.82)	133 (65.20)	0.285
	Female	31 (28.18)	71 (34.80)	
Diagnosis, no. (%)	SLD	0 (0.00)	4 (1.95)	0.34
	5q-	1 (0.92)	0 (0.00)	
	MLD	24 (22.02)	45 (21.95)	
	EB-1	37 (33.94)	60 (29.27)	
	EB-2	40 (36.70)	87 (42.44)	
	MDS/MPN, MDS-U	7 (6.42)	9 (4.39)	
Ferritin, ng/ml, median (range)	Continuous variable	1198.15 (11.4-10022)	974.1 (36.5-18038.7)	0.107
PTR before transplantation, no. (%)	Yes	41 (67.06)	27 (26.47)	<0.001
	No	23 (35.94)	75 (73.53)	
Donor age, years, median (range)	Continuous variable	40 (9-59)	36 (10-61)	0.757
Donor source, no. (%)	MSD	55 (50.00)	88 (42.93)	0.342
	HID	48 (43.64)	107 (52.20)	
	URD	7 (6.36)	10 (4.88)	
Donor–recipient relationship, no. (%)	Siblings	64 (58.18)	110 (53.66)	0.408
	Off-siblings	29 (26.36)	66 (32.20)	
	Parents	6 (5.45)	16 (7.80)	
	Others	11 (10.00)	13 (6.34)	
Donor-patient sex match, no. (%)	Female to Male	46 (42.59)	62 (30.24)	0.115
	Male to Female	25 (23.15)	50 (24.39)	
Donor-patient sex match, no. (%)	Male to Female	26 (24.07)	72 (35.12)	0.115
	Female to Female	11 (10.19)	21 (10.24)	
Blood types of donors to recipients, no. (%)	Matched	56 (50.91)	112 (54.63)	0.295
	Major mismatched	29 (26.36)	46 (22.44)	
	Minor mismatched	20 (18.18)	28 (13.66)	
	Major and minor mismatched	5 (4.55)	19 (9.27)	
Conditioning regimen, no. (%)	Without decitabine	37 (33.64)	63 (30.88)	0.709
	With decitabine	73 (66.36)	141 (69.12)	
Mononuclear cells, ×10^8^/kg, median (range)	Continuous variable	10.4 (4.4-26.4)	10.6 (5.2-27.7)	0.689
CD34^+^cells, ×10^6^/kg, median (range)	Continuous variable	2.7 (1.5-8)	2.9 (1.2-8.6)	0.184
Follow-up of alive patients, months, median (range)	Continuous variable	32 (6-126)	24 (5-160)	0.052

### Clinical outcomes

**Figure 1:**
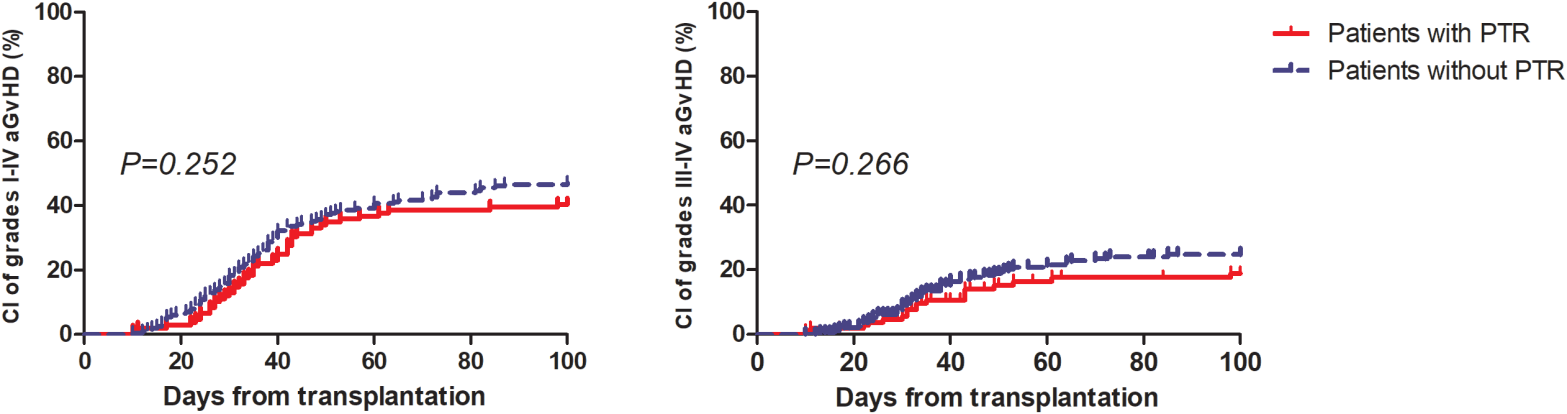
Cumulative incidence of grade I to IV aGvHD and III to IV aGvHD among patients with and without PTR.

The major clinical outcomes of the patients are shown in **Table 2**. More patients in the PTR group experienced febrile neutropenia (40.37% vs. 23%, *P*=0.002), but the rates of bloodstream infection before engraftment (16.36% vs. 9.27%, *P*=0.093) and CMV reactivation (40% vs. 48.3%, *P*=0.157) were similar between the two groups. Only one patient experienced primary graft rejection and PTR. Eventually, 84 out of 110 patients (76.4%) with PTR achieved platelet engraftment at day 28, while 181 out of 205 patients (88.3%) without PTR achieved platelet engraftment at day 28 (*P*=0.007). The median days of neutrophil and platelet engraftment were one and two days slower among patients in the PTR group, respectively, than among patients without PTR (*P*<0.001). No differences were observed in the CI of 100-day grades I to IV and III to IV aGvHD between the two groups (**Figure 1**). Until the last follow-up, patients in the PTR group received significantly more red blood cell (RBC) and platelet (PLT) transfusions. The median units of RBC and PLT transfusions were 17 (range: 2-143.5) and 13 (range: 3-85) in the PTR group versus 10 (range: 0-77.5) and 7 (range: 1-75) in the non-PTR group (*P*<0.001). Among the 68 patients with PTR pre-HSCT, only 28 of 52 (53.8%) patients who received rabbit anti-thymocyte globulin (rATG) or porcine anti-lymphocyte globulin (pALG) during conditioning experienced PTR post-HSCT compared to 13 of 16 (81.3%) patients who did not receive rATG or pALG (*P*=0.05) suffered PTR post-HSCT.

**Table 2:** Major clinical outcomes of patients.

Variables	Patients with PTR (N=110)	Patients without PTR (N=205)	*P* value
Febrile neutropenia, no. (%)	44 (40.37)	46 (23.00)	0.002
Bloodstream infection, no. (%)	18 (16.36)	19 (9.27)	0.093
CI of 100-day of I-IV aGvHD (%) (95%CI)	40.4 (29.4-51)	47.0 (39.6-57.1)	0.252
CI of 100-day of III-IV aGvHD (%) (95%CI)	18.9 (7-35.2)	24.8 (15.1-35.7)	0.266
Neutrophil engraftment, days, median (range)	14 (9-23)	13 (9-23)	<0.001
Platelet engraftment, days, median (range)	17 (8-28)	15 (7-28)	<0.001
Percentage of platelet engraftment on 28-day	84 (76.4)	181 (88.3)	0.007
CMV reactivation, no. (%)	44 (40)	99 (48.3)	0.159
RBC transfusions post-HSCT, units, median (range)	18 (2-143.5)	10 (0-77.5)	<0.001
PLT transfusions post-HSCT, units, median (range)	13 (3-85)	7 (1-75)	<0.001

CI cumulative incidence, aGvHD acute graft versus host disease, cGvHD chronic graft versus host disease, RBC red blood cell, PLT platelet.

In multivariate analysis (**Table 3**), PTR post-HSCT in one-month, male sex, and bloodstream infection were independent adverse risk factors of neutrophil engraftment, while PTR post-HSCT at one month, haploidentical donor, and ferritin>1041ng/ml pre-HSCT were independently associated with poor platelet engraftment.

**Table 3:** Univariate and multivariate analysis of hematopoietic recovery.

Variables	Factors	Comparison	Neutrophil engraftment				Platelet engraftment			
			Univariate analysis		Multivariate analysis		Univariate analysis		Multivariate analysis	
			HR (95%CI)	*P* value	HR (95%CI)	*P* value	HR (95%CI)	*P* value	HR (95%CI)	*P* value
Donor source	HID	MSD	0.8 (0.63-1.01)	0.06	0.94 (0.72-1.24)	0.682	0.67 (0.52-0.86)	0.002	0.64 (0.5-0.83)	<0.001
	URD		0.79 (0.47-1.31)	0.36	1.76 (0.86 - 3.57)	0.12	0.74 (0.43-1.29)	0.287	0.97 (0.53-1.78)	0.927
Patient age	–	CV	1 (0.99-1.01)	0.689	–	–	0.99 (0.98-1.01)	0.283	–	–
Patient gender	Female	Male	1.34 (1.05-1.7)	0.017	1.37 (1.07 - 1.75)	0.014	1.37 (1.06-1.78)	0.015	1.08 (0.64-1.81)	0.777
Donor-patient sex match	M to F	F to M	1.18 (0.87-1.59)	0.282	–	–	1.49 (1.08-2.06)	0.015	1.4 (0.83-2.37)	0.209
	M to M		1.11 (0.84-1.46)	0.456	–	–	1.1 (0.81-1.48)	0.545	1.03 (0.76-1.4)	0.85
	F to F		1.36 (0.91-2.02)	0.13	–	–	1.27 (0.83-1.94)	0.271	1.03 (0.55-1.95)	0.921
Blood types of donors to recipients	Major	Matched	1.1 (0.84-1.45)	0.49	–	–	1.05 (0.78-1.41)	0.738	–	–
	Minor		0.87 (0.63-1.21)	0.415	–	**-**	0.78 (0.54-1.12)	0.177	–	–
	Major and minor		0.87 (0.57-1.34)	0.526	–	–	1.37 (0.88-2.14)	0.159	–	–
Donor age	–	CV	1.01 (1-1.02)	0.073	1.10 (1 - 1.02)	0.151	1 (0.99-1.01)	0.589	–	–
Ferritin(>1041ng/ml)	–	≤1041ng/ml	0.8 (0.64-1)	0.049	0.85 (0.68 - 1.07)	0.169	0.75 (0.59-0.95)	0.019	0.72 (0.57-0.92)	0.01
Amount of MNC	–	CV	1.01 (0.98-1.04)	0.444	–	–	0.96 (0.92-0.99)	0.018	0.97 (0.94-1.01)	0.168
Amount of CD34^+^ cells	–	CV	0.96 (0.88-1.05)	0.385	–	–	1.02 (0.93-1.12)	0.684	–	–
Without PTR	–	with PTR	1.72 (1.36-2.18)	<0.001	–	–	1.76 (1.36-2.29)	<0.001	1.74 (1.33-2.28)	<0.001
With BSI	–	Without BSI	0.56 (0.4-0.8)	0.001	0.63 (0.44-0.91)	0.012	0.6 (0.4-0.91)	0.017	0.68 (0.45-1.04)	0.074

HID haploidentical donor, URD unrelated donor, MSD matched sibling donor, CV continuous variable, F female, M male, MNC mononuclear cells, PTR platelet transfusion refractoriness, BSI bloodstream infection.

### Survival and primary cause of death

**Figure 2:**
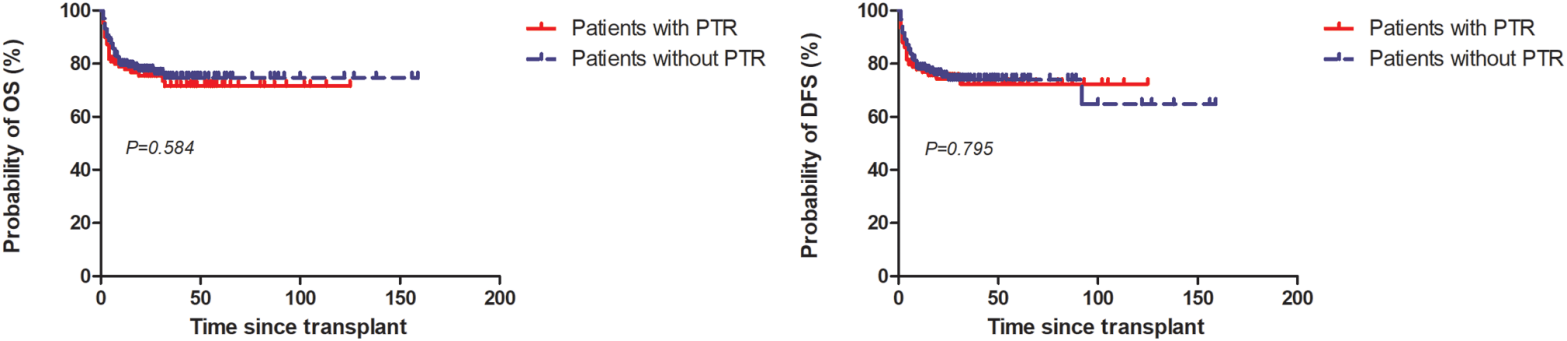
Overall survival (OS) and disease-free survival (DFS) among patients with and without PTR.

At 5 years, the estimated OS and DFS rates in patients with PTR were 71.6% (95%CI: 61-79.9) and 71.7% (95%CI: 61.6-79.7), respectively, in contrast to 74.7% (95%CI: 67.1-80.8) (*P*=0.584) and 74.1% (95%CI: 66.9-79.9) (*P*=0.679) in patients without PTR (**Figure 2**).

A total of 74 deaths had occurred at the last follow-up. The primary COD values are listed in **Table 4**. Strikingly, fatal hemorrhages and all hemorrhagic events in one month post-HSCT between the two groups were similar (**Supplementary Table S2**).

**Table 4:** Primary causes of death among patients.

COD	Patients with PTR (n=28) (%)	Patients without PTR (n=46) (%)	*P* value
aGvHD	10 (35.7)	24 (52.2)	0.674
Infection	7 (25)	10 (21.7)	
cGvHD	2 (7.1)	3 (6.5)	
Relapse	5 (17.9)	7 (15.2)	
Intracranial hemorrhage	2 (7.1)	1 (2.2)	
Secondary graft failure	1 (3.6)	1 (2.2)	
Renal failure	1 (3.6)	–	

aGvHD acute graft versus host disease, cGvHD chronic graft versus host disease.

## Discussion

To the best of our knowledge, this is the first retrospective study with a large sample size to evaluate the effects of post-HSCT PTR before engraftment in patients with MDS or MDS/MPN. Our study showed PTR in one-month post-HSCT did not influence the survival of these patients, nor did the incidence of aGvHD. However, it delayed the engraftment of neutrophils and platelets and significantly decreased the rate of platelet engraftment on day 28.

In accordance with previous studies, we found PTR in one-month post-HSCT did not influence the survival of patients with MDS. Tan et al. evaluated the impact of PTR on survival before and after cord blood cell transplantation (CBT). In contrast to patients without PTR, they identified that only PTR 31–45 days after CBT, instead of PTR before or after HSCT in one month, was significantly associated with inferior survival (6). This finding was also consistent with another study from Spain that demonstrated PTR in one-month post-HSCT could significantly decrease the survival rate of patients undergoing peripheral blood stem cell (PBSC) transplantation but marginally influenced the survival of patients receiving UCB (5). In the MDS-specific setting of our study, we applied PBSC as the main stem cell source and focused on PTR from stem cell infusion to engraftment to minimize the effect of virus reactivation and aGvHD, most of which occurred one month later. However, other studies have found that patients with PTR have significantly inferior survival compared to those without PTR (2, 4, 18). These differences may be partially explained by the different hematological diseases analyzed, treatment procedures, and study periods.

In addition, we did not observe that patients in the PTR group experienced higher rates of aGvHD, BSI, or fatal hemorrhagic complications. A major concern with PTR is the potentially increased risk of bleeding. However, our findings contradicted this hypothesis. This may be due to recent improvements in supportive care, such as high-efficacy anti-infection prophylaxis, matched transfusion techniques, and the application of thrombopoietin receptor agonists (19–22). Moreover, delayed successful platelet engraftment may counteract the negative effects of early PTR. In our study, although only 76.4% of patients with PTR achieved platelet engraftment on day 28, most of the remaining patients became platelet independent at approximately 40 days post-HSCT.

Indeed, PTR post-HSCT at one month was independently associated with slower platelet engraftment in our study. This finding is consistent with that of another study (6). They identified patients suffering from PTR 0–15 days after CBT as an independent adverse factor in achieving neutrophil engraftment, and PTR 0–30 days after CBT as an independent poor factor in achieving platelet recovery. In addition, we found a higher rate of platelet non-engraftment on day 28 among patients in the PTR group, which is consistent with the findings of another study (23). However, the time to platelet engraftment was similar between the two groups.

We also found that haploidentical transplantation and ferritin>1041ng/ml (median level in our study) were independent risk factors for platelet engraftment. Raj et al. reported that compared to patients with MSD, patients with HID had lower and slower recovery of platelets. The median number of days to platelet engraftment was 14 (95%CI: 14-15) and 28 (95%CI: 27-31), respectively (24). A Chinese study also demonstrated a lower rate of platelet engraftment on day 100 after haploidentical transplantation, in contrast to that in MDS patients (25). High pre-HSCT ferritin levels could increase the rate of infection and impair the function of progenitors by reactive oxygen species. Malki et al. reported that iron overload characterized by a high ferritin level of >2000ng/ml in UCB could lead to delayed platelet engraftment. At 3 months, the CI of platelet engraftment in patients with ferritin levels of >2000 ng/ml was 52% (95%CI: 0.29-0.71) compared to 81% (95%CI:0.69-0.88) in patients with ferritin ≤2000 ng/ml (*P*=0.044) (26).

Our study has some limitations. First, as a retrospective study, selection bias is unavoidable; for instance, we may prefer HLA-matched or cross-matched platelets in patients with PTR, which increases the transfusion effect and improves survival. Second, for those with PTR, we did not uniformly perform tests to further identify the reasons. We descried the clinical conditions such as febrile neutropenia (FN), BSI, and bleeding related with PTR in details in **Table 1**, **Supplementary Table S2**, respectively. We noticed that FN and BSI were significantly and marginally different between the two groups while bleeding events was similar between them. Therefore, massive platelet consumption caused by infection and drug interaction may also cause PTR post-HSCT (6). Based on our research, our recommendation is to perform anti-class I human leukocyte Antigens (HLA), anti-human platelet antigens (HPA), anti-membrane glycoproteins, and anti-36 antibodies among patients with PTR to differentiate immunologic from non-immunologic factor and guider treatment further (27).

Taken together, our study indicates that among patients with MDS or MDS/MPN, PTR post-HSCT in one-month could impair hematopoietic recovery but not survival. Future studies should explore the underlying mechanisms and interventions for this phenomenon.

## Data Availability

The raw data supporting the conclusions of this article will be made available by the authors, without undue reservation.
