# Supplementary material for: Impact of platelet transfusion refractoriness in the first 30 days post-hematopoietic stem cell transplantation on outcomes of patients with myelodysplastic syndrome
Source: Front Immunol. 2024 Sep 25;15:1437176. doi: 10.3389/fimmu.2024.1437176 (PMC11461267; doi:10.3389/fimmu.2024.1437176)
Supplement: Supplementary file 1 [file Table1.docx]

**Supplementary Table 1 Details of donor types, conditioning regimens, and GVHD prophylaxis**

| Donor types | Conditioning regimen A | Conditioning regimen B | GVHD prophylaxis |
| --- | --- | --- | --- |
| Haploidentical donor | busulfan 3.2 mg/kg/day for three days, cyclophosphamide 40 mg/kg/day for two days, fludarabine 30 mg/m^2^/day for three days, and cytarabine 2-4g/m^2^/day at divided dose for three days | busulfan 3.2 mg/kg/day for three days, cyclophosphamide 40 mg/kg/day for two days, cladribine at 5mg/m^2^/day for three days, and idarubicin at 12 mg/m^2^/day for three days. | rabbit anti-thymocyte globulin (ATG) at 2.5 mg/kg/day of 4 days, low-dose methotrexate (MTX, 15 mg/m^2^ on day +1 and 10 mg/m^2^ on days +3, +6, and +11), mycophenolate mofetil (MMF), and tacrolimus or cyclosporine |
| Matched unrelated donor |  |  |  |
| Matched related donor |  |  | low-dose methotrexate (MTX, 15 mg/m^2^ on day +1 and 10 mg/m^2^ on days +3, +6, and +11), mycophenolate mofetil (MMF), and tacrolimus or cyclosporine |

Notes: 5-day decitabine could be added to the conditioning A or B.
