# Supplementary material for: Impact of platelet transfusion refractoriness in the first 30 days post-hematopoietic stem cell transplantation on outcomes of patients with myelodysplastic syndrome
Source: Front Immunol. 2024 Sep 25;15:1437176. doi: 10.3389/fimmu.2024.1437176 (PMC11461267; doi:10.3389/fimmu.2024.1437176)
Supplement: Supplementary file 2 [file Table2.docx]

**Supplementary Table 2 Bleeding events between two groups**

| **Variables** | **Patients with PTR**  **(N=110)** | **Patients without PTR**  **(N=205)** | ***P* value** |
| --- | --- | --- | --- |
| Gastrointestinal tract bleeding, no. (%) | 12 (10.9) | 16 (7.8) | 0.291 |
| Vaginal bleeding, no. (%) | 4 (3.6) | 5 (2.4) |  |
| Urinary tract bleeding, no. (%) | 8 (7.3) | 9 (4.4) |  |
| Respiratory tract bleeding, no. (%) | 1 (0.9) | 1 (0.5) |  |
| Gastrointestinal and urinary tract bleeding, no. (%) | 1 (0.9) | 4 (2.0) |  |
| Respiratory and urinary tract bleeding, no. (%) | 1 (0.9) | 0 |  |
| Gastrointestinal tract and fundus bleeding, no. (%) | 0 | 2 (1) |  |
| Urinary tract and fundus bleeding, no. (%) | 1 (0.9) | 0 |  |
| Brian and fundus bleeding, no. (%) | 1 (0.9) | 0 |  |
| Orval cavity bleeding | 0 | 2 (1) |  |
| Gastrointestinal, urinary tract, and vaginal bleeding, no. (%) | 1 (0.9) | 0 |  |
| Nasal and urinary tract bleeding | 1 (0.9) | 0 |  |
| Nasal and gastrointestinal tract bleeding | 0 | 3 (1.5) |  |
